# Exploring the Role of Amino Acid-Derived Multivariate Metal–Organic Frameworks as Catalysts in Hemiketalization Reactions

**DOI:** 10.1021/acs.inorgchem.3c00495

**Published:** 2023-04-28

**Authors:** Cristina Negro, Sergio Sanz-Navarro, Antonio Leyva-Pérez, Donatella Armentano, Jesús Ferrando-Soria, Emilio Pardo

**Affiliations:** † Instituto de Ciencia Molecular (ICMol), Universidad de Valencia, 46980 Valencia, Spain; ‡ Instituto de Tecnología Química (UPV−CSIC), Universidad Politècnica de València−Consejo Superior de Investigaciones Científicas, Avda. de los Naranjos s/n, 46022 Valencia, Spain; § Dipartimento di Chimica e Tecnologie Chimiche (CTC), Università della Calabria, Rende 87036, Italy

## Abstract

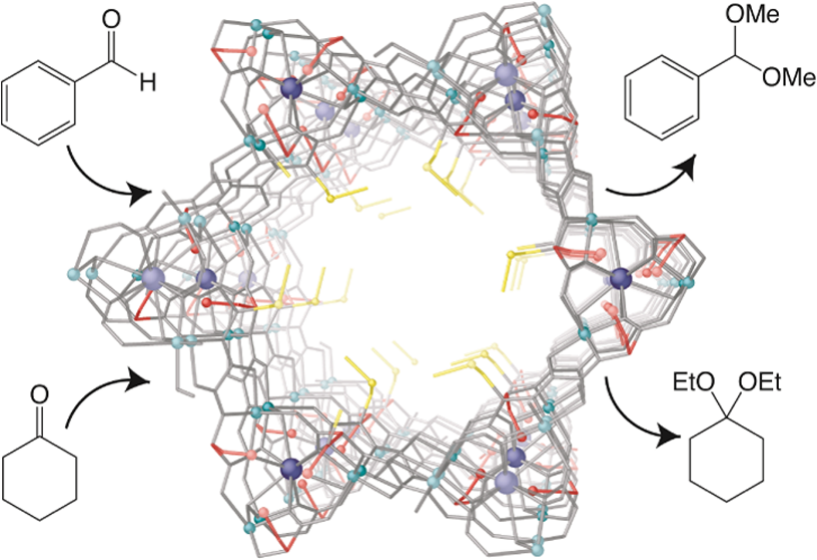

Understanding the host–guest chemistry in MOFs represents a research field with outstanding potential to develop in a rational manner novel porous materials with improved performances in fields such as heterogeneous catalysis. Herein, we report a family of three isoreticular MOFs derived from amino acids and study the influence of the number and nature of functional groups decorating the channels as a catalyst in hemiketalization reactions. In particular, a multivariate (MTV) MOF **3**, prepared by using equal percentages of amino acids L-serine and L-mecysteine, in comparison to single-component (“traditional”) MOFs, derived from either L-serine or L-mecysteine (MOFs **1** and **2**), exhibits the most efficient catalytic conversions for the hemiketalization of different aldehydes and ketalization of cyclohexanone. On the basis of the experimental data reported, the good catalytic performance of MTV-MOF **3** is attributed to the intrinsic heterogeneity of MTV-MOFs. These results highlight the potential of MTV-MOFs as strong candidates to mimic natural nonacidic enzymes, such as glycosidases, and to unveil novel catalytic mechanisms not so easily accessible with other microporous materials.

## Abstract 2

The influence of the number and nature of distinct functional groups decorating MTV-MOF pores in the catalysis of hemiketalization reactions was investigated.

## Introduction

Multivariate metal–organic frameworks (MTV-MOFs)^1−7^ represent a novel generation of porous crystalline materials within MOFs, constituted of linkers with identical backbones but different functionalities decorating the ligand, where heterogeneity and complexity of composition have opened the way to synergetic behaviors and thus to novel and/or enhanced properties compared with single-component (“traditional”) MOFs.^8−10^ Nevertheless, it is difficult to obtain precise atomic information on the location and distribution of the different functionalities decorating the MTV-MOF channels, and further work adapting/improving existing characterization techniques or implementing new ones will be needed.^11−13^ The exciting properties exhibited by MTV-MOFs in different applications overcome this uncertainty, and it has not been a limitation for the growing interest in developing novel MTV-MOFs.^14−19^

Elegant application examples of taking advantage of the heterogeneity of MTV-MOFs have been reported for heterogeneous catalysis.^20−23^ Indeed, such complex porous platforms are a step closer to engendering materials that mimic the existing complexity and functionality of enzymes.^24,25^ A beautiful example is the seminal work reported by Fracaroli et al.,^20^ where, in an MTV-MOF, up to seven postsynthetic modifications were performed to lead to more complex MTV-MOFs with channels decorated with a tripeptide. In particular, the one with the H_2_N-Cys-His-Asp-CONHL sequence (Cys = cysteine, His = histidine, Asp = aspartic acid, and L = organic backbone) of the endopeptidase enzyme tobacco etch virus (TEV) was investigated as a mimic of this enzyme for the sequence-specific bond cleavage of a pentapeptide. Despite the low conversion achieved, this work illustrated that it is possible to transfer the compositional heterogeneity of MTV-MOFs into a complex target application.

One of our main research lines has been devoted to exploring the development of MTV-MOFs with oxamidato-based ligands derived from amino acids, with the main aim to take advantage of the heterogeneity of composition and the intrinsic flexibility of the residues decorating the MTV-MOF channels toward more complex applications. So far, we have observed a successful transfer from the complexity of composition to the improvement of performance in water remediation.^14,18,26,27^ In particular, an MTV-MOF {Ca^II^Cu_6_^II^[(*S*,*S*)-methox]_1.43–1.46_[(*S*,*S*)-serimox]_1.57–1.54_(OH)_2_(H_2_O)}·30H_2_O (where methox = bis[(*S*)-methionine]oxalyl diamide and serimox = bis[(*S*)-serine]oxalyl diamide) with functional channels decorated with approximately 50% of hydroxyl groups and 50% thioalkyl ones derived from natural amino acids L-serine and L-methionine, respectively, has proven very efficient in the simultaneous capture of contaminants with inorganic and organic nature.^14^ Also, this has been further validated with an isoreticular MTV-MOF {Sr^II^Cu_6_^II^[(*S*,*S*)-methox]_1.50_[(*S*,*S*)-mecysmox]_1.50_(OH)_2_(H_2_O)}·36H_2_O (where methox = bis[(*S*)-methylcysteine]oxalyl diamide), which outperforms single-component MOFs in the removal of emergent neonicotinoid contaminants from water.^18^

On this basis and with the background inspiration of the active centers of enzymes, where different organic functionalities are able to act synergistically to interact, adapt, and efficiently catalyze the transformation of specific substrates, the aim of this investigation has been to explore within a family of isoreticular MOFs the role and influence of the presence and absence of functional channels decorated with more than one distinct amino acid residue in catalysis. To this end, we have focused on the use of two previously reported MOFs, with formulas {Sr^II^Cu_6_^II^[(*S*,*S*)-serimox]_3_(OH)_2_(H_2_O)}·38H_2_O (**1**)^28,29^ and {Sr^II^Cu_6_^II^[(*S*,*S*)-mecysmox]_3_(OH)_2_(H_2_O)}·15H_2_O (**2**),^30^ and a novel MTV-MOF of formula {Sr^II^Cu_6_^II^[(*S*,*S*)-serimox]_1.50_[(*S*,*S*)-mecysmox]_1.50_(OH)_2_(H_2_O)}·12H_2_O (**3**) (Scheme S1 and Figure S1) as catalysts for hemiketalization and ketalization reactions.

The addition of alcohols to the carbonyl group of aldehydes and ketones is an essential reaction in nature^31^ and industrial synthesis^32^ since the electronic density of the carbonyl group is completely switched from electron-rich to electron-poor. Hemiketalization and ketalization reactions have been traditionally used in organic synthesis to protect the aldehyde and ketone groups during other reactions, which are regenerated after simple water-acidic treatment.^33^ However, the acid-catalyzed conditions required for hemiketalization and ketalization reactions are not always compatible with other groups. Indeed, it is not surprising to find in the literature synthetic routes where a functional group is previously protected by other methodologies before the carbonyl group is protected under acid-catalyzed conditions.^34^ Thus, any catalytic method for hemiketalization and ketalization reactions based on nonacidic conditions is of interest.

Nature makes use of nonacid enzymes, such as glycosidases, to catalyze hemiketalization and ketalization reactions by the cooperative action of two different pocketed base groups, *i*.*e*., carboxylates.^35^ In this context, we have recently reported that MOF **1** is able to selectively hydrolyze ketal groups in complex organic molecules under nonacid conditions, by the combined action of the alcohol groups from serine residues within the MOF channels in the presence of water.^36^ Since ketalization reactions are reversible, we hypothesized that the same MOF **1** could catalyze the formation of ketal under anhydrous conditions. Unfortunately, this reaction only occurred to a very minor extent.^35^ Thus, at this point, on the basis of the efficient σ-hole interactions observed on MOFs containing thioether residues on the recognition and capture of organic contaminants,^18^ as well as the fact that cyclic thiols in stoichiometric amounts, after forming dithianes, have been traditionally used to protect carbonyl groups,^37^ we turned our attention to MOF **2**. However, despite these a priori optimal conditions, **2** has not been revealed as a very efficient catalyst. Intrigued by these results, we aimed to take advantage of the heterogenization and synergistic effect observed in MTV-MOFs, to explore the possibility that the MTV-MOF **3** could be active for hemiketalization and ketalization reactions, since the combination of −CH_2_OH and −CH_2_SCH_3_ groups in **3** could help in carbonyl group activation. Thus, the amino acid residues containing alcohol and thioether groups in MTV-MOF **3** will not form permanent bonds but could cooperatively activate the carbonyl group in the presence of external alcohol, in a way similar to glycosidases. The catalytic mechanism of this microporous bifunctional solid will significantly differ from previous examples with microporous solids, *i*.*e*., zeolites^38,39^ and MOFs,^40^ based on amine/acid and other functional groups. We present below that this strategy works and that MTV-MOF **3** catalyzes hemiketalization and ketalization reactions within its confined space more efficiently than MOFs **1** and **2**.

## Results and Discussion

### Synthesis and Characterization

In this work, we show an expansion of the application of the metalloligand design strategy to synthesize a novel water-stable tridimensional (3D) MTV-MOF, with formula {Sr^II^Cu_6_^II^[(*S*,*S*)-serimox]_1.50_[(*S*,*S*)-mecysmox]_1.50_(OH)_2_(H_2_O)}·12H_2_O (**3**), obtained as green prisms by slow diffusion in H-shaped tubes at room temperature of aqueous solutions containing stoichiometric amounts of an equimolar mixture of (Me_4_N)_2_{Cu_2_[(*S*,*S*)-serimox](OH)_2_}·5H_2_O and (Me_4_N)_2_{Cu_2_[(*S*,*S*)-mecysmox](OH)_2_}·5H_2_O in one arm and Sr(NO_3_)_2_ in the other.

**Figure 1:**
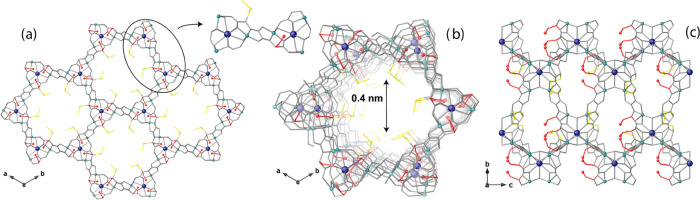
(a) View of the porous structure of **3** along the *c* crystallographic axis (inset emphasizes the dianionic bis(hydroxo) dicopper(II) building blocks). (b, c) Views of a single channel for the porous structure of MTV-MOF **3** along the *c* (b) and *a* (c) axes (the crystallization water molecules are omitted for clarity). Copper(II) and strontium(II) ions from the network are represented as cyan and blue spheres, respectively. Oxygen and sulfur atoms from the residues are shown as red and yellow spheres, respectively. The organic ligands are represented as gray sticks, with the exception of L-serine (−CH_2_OH) and L-methylcysteine (−CH_2_SCH_3_) residues, which are represented as red and yellow sticks, respectively.

The crystal structure of **3** determined by single-crystal X-ray diffraction (SCXRD) consists of a chiral honeycomb-like 3D strontium (II)/copper (II) network featuring functional hexagonal channels of approximately 0.4 nm, developing along the *c* crystallographic axis, where the methylenethiomethyl chains of methylcysteine derivatives are located (Figure 1). It is an **acs** uninodal sixfold-connected net (4^9^.6^6^), built from trans-oxamidato-bridged dicopper(II) units of {Cu_2_^II^[(*S*,*S*)-serimox]} and {Cu_2_^II^[(*S*,*S*)-mecysmox]} (Scheme S1 and Figures 1 and S1–S3), which are expected to be statistically disordered in the crystal structure. They act as linkers between the Sr^II^ ions through carboxylate groups (Figure 1a,b). Neighboring Cu^2+^ and Cu^2+^/Sr^2+^ ions are auxiliary interconnected, in a μ_3_ fashion, by aqua/hydroxo groups (in a 1:2 statistical distribution) (inset Figure 1a). As previously tested for the refinement of best model in the crystal structure of the isoreticular MTV-MOF {Ca^II^Cu_6_^II^[(*S*,*S*)-methox]_1.43-1.46_[(*S*,*S*)-serimox]_1.57-1.54_(OH)_2_(H_2_O)}·30H_2_O,^14^ a similar percentage of mixed serimox and mecysmox (confirmed by the composition analysis, vide infra C, H, S, N in the Experimental Section and Supporting Information) lead to superimposed snapshots of mixed {Cu_2_^II^[(S,S)-mecysmox/serimox]} metalloligands. Thus, once more, the disorder in **3** has been modeled as an averaged view of the crystal structure, which is the spatial average of all molecule/fragment structures in the crystal *via* only one unit cell (see crystallographic details in the Supporting Information). This, even if it could seem counterintuitive, it is a better description of the real situation, where pure copper(II) metalloligands of each amino acid-derived ligand are randomly distributed (with 1:1 ratio) within the net (see crystallographic details in the Supporting Information). Looking at such modeled crystal structure of **3**, the two “arms” decorated with serine and methylcysteine derivatives, respectively, show different orientations, with the methylcysteine one being more distended, within the largest octagonal pores, and the serine one bent and confined in the smallest channels developing along the *a* crystallographic axis (Figures 1b,c and S3). Here, it is worth noting that MTV-MOFs, as already underlined in our previous works,^14,18,26,27^ show as a thrilling feature an impressive flexibility of the functional arms confined within the pores, exhibiting different conformations depending on the target molecules captured in pores. Thus, loaded reactants of a given catalytic reaction might have an effect on the final conformation adopted by the flexible aminoacidic arms decorating the pores. Indeed, it depends on host–guest interactions, which are at the origin of the stabilization of such species within confined spaces.

Finally, the estimated empty volume for **3**, without the crystallization water molecules, is 1764.7 (1) Å^3^, which represents 48.3% of potential void per unit cell volume [*V* = 3653.9 Å^3^].

In addition to the structural characterization and elemental analysis, the nature and identity of MTV-MOF **3** were also established by powder X-ray diffraction (PXRD), N_2_ adsorption isotherm at 77 K, and thermogravimetric analyses (TGAs) (see the Supporting Information). The experimental PXRD pattern of **3** is consistent with the theoretical one, extracted from SCXRD (Figure S4), which confirms the homogeneity and purity of the powder sample. The permanent porosity of **3** was supported by measuring the N_2_ adsorption isotherm at 77 K and compared to the adsorption isotherms of traditional MOFs **1** and **2** (Figure S5). They confirm the permanent porosity for all three materials, with N_2_ adsorbed amounts for **3** between the ones of **1** and **2**, but closer to **2**, which is consistent with the accessible void spaces shown in the crystal structures. The solvent content of **3** was established by TGA (Figure S6), with a calculated percentage weight loss value of 85% at 388 K, which corresponds to 12 water molecules. **3** is stable up to 200 °C when decomposition starts.

### Catalytic Studies

**Figure 2:**
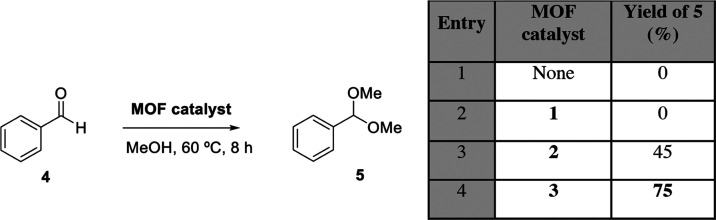
Results for the MOF-catalyzed hemiketalization reaction of benzaldehyde **4** with methanol. Selectivity to **5** is 100% (conversion equals yield). GC yields.

Figure 2 shows the catalytic results for the hemiketalization reaction of benzaldehyde **4** with methanol. After screening some reaction conditions, the best results were obtained at 60 °C after an 8 h reaction time. Under these reaction conditions, it can be seen that MOF **1** is completely inactive for the reaction (entries 1 and 2), as observed before,^36^ and that MOF **2** shows some activity, to afford a 45% yield of hemiacetal **5** (entry 3). In contrast, MTV-MOF **3** catalyzes the reaction much more efficiently to afford product **5** in 75% yield (entry 4). The selectivity of the reaction is complete since other products are not observed, and the fact that the transformation does not advance further is probably due to reaching equilibrium. Magnesium sulfate was added to the reaction media in order to adsorb the water formed during the reaction, further shifting the equilibrium toward the hemiketal product **5**. However, the addition of MgSO_4_ did not improve the yield of **5**, since the generated water is probably retained in the highly polar MOF pores and reacts back with **5** at high conversions of **4**.

In order to confirm the retention of the structural integrity of **1**–**3** under catalytic conditions, as well as to rule out the possibility of partial/total structural collapse in **1** and **2**, N_2_ adsorption isotherms at 77 K and PXRD were also performed after catalytic experiments (Figures S5b and S7). From PXRD, it can be concluded that all materials are robust enough to maintain their structural integrity under reaction conditions. In an analogous way, the similarity of Brunauer–Emmett–Teller (BET)^41^ surface areas before [791(**1**), 633(**2**), and 682(**3**) m^2^/g] and after [763(**1**), 609(**2**), and 670(**3**) m^2^/g] catalysis support the retention of porosity and further reflect the stability of **1**–**3**. The stability of MTV-MOF **3** in aqueous and nonaqueous solvents was further validated by PXRD (Figure S8) of polycrystalline samples of **3** immersed for 24 h in hot water, dimethylformamide, methanol, and acetonitrile.

**Figure 3:**
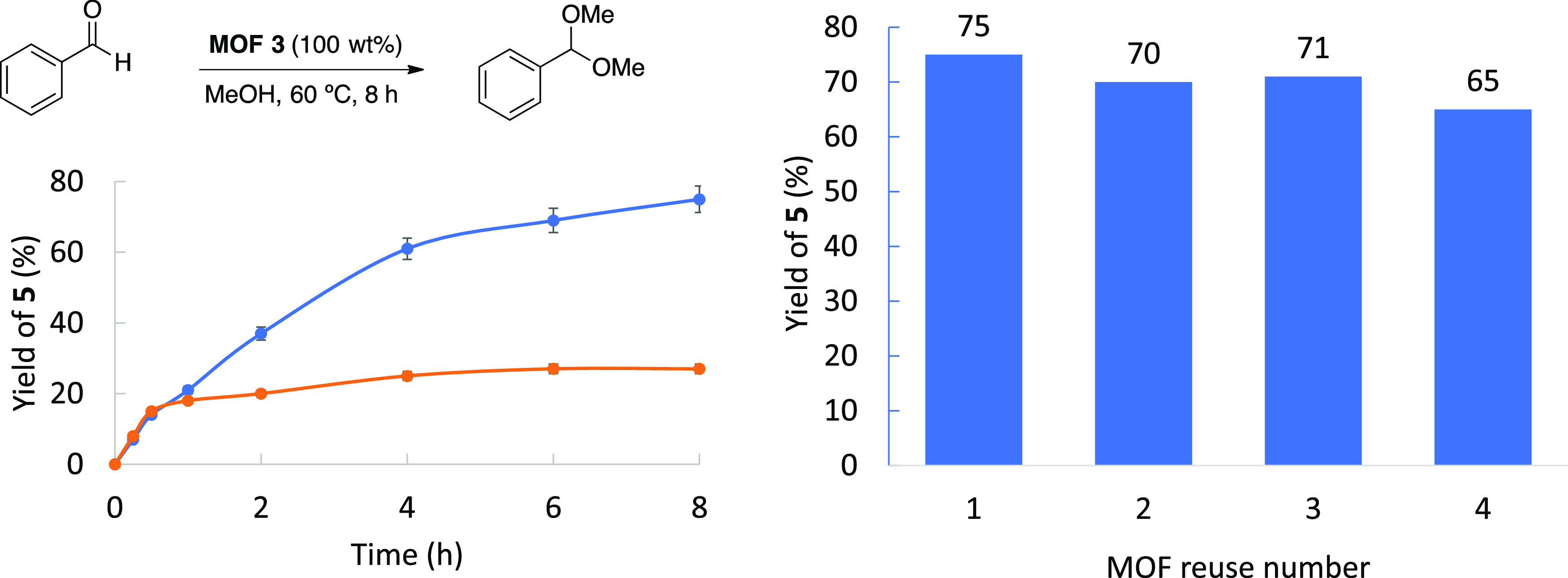
(Left) Hot filtration test for MTV-MOF **3** during the hemiketalization reaction of benzaldehyde **4** with MeOH under optimized reaction conditions. Error bars account for a 5% uncertainty. (Right) Reuse of MTV-MOF **3** under the same reaction conditions. GC yields.

Figure 3 (left) shows the hot filtration test for the reaction catalyzed by the MTV-MOF **3**, where it can be seen that the hemiketalization reaction of benzaldehyde **4** stops after the solid catalyst is filtered off. In accordance, Figure 3 (right) shows that MTV-MOF **3** could be reused up to 3 times without appreciable depletion of the final yield of **5**.

**Figure 4:**
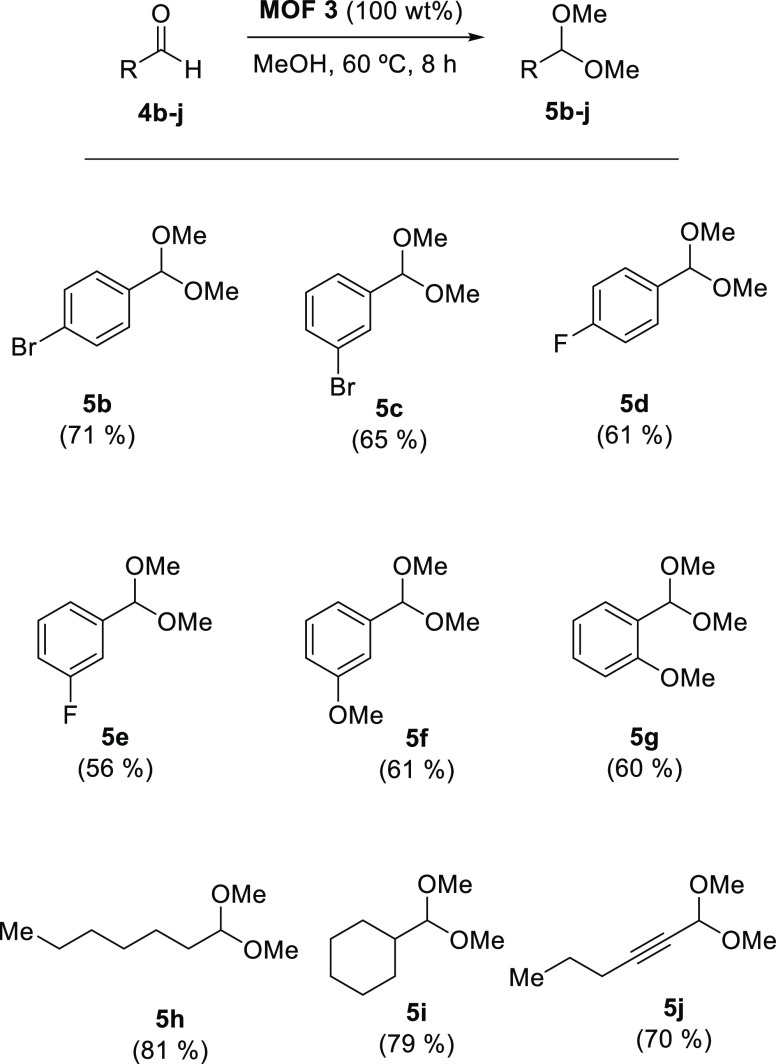
Scope of aldehydes for the hemiketalization reaction catalyzed by MTV-MOF **3**. GC yields.

The scope of aldehydes was then assessed for the hemiketalization reaction catalyzed by MTV-MOF **3**, under the optimized reaction conditions. The results are shown in Figure 4. It can be seen that aromatic aldehydes with halide and methoxy substituents in different positions of the aryl ring can be obtained in good yields (products **5b–g**) and that alkyl aldehydes are even better reactive (products **5h–i**), including acid-sensitive propargyl aldehydes (**5j**). These results support the ability of MTV-MOF **3** to catalyze the hemiketalization reaction of aldehydes with disparate electronics.

**Figure 5:**
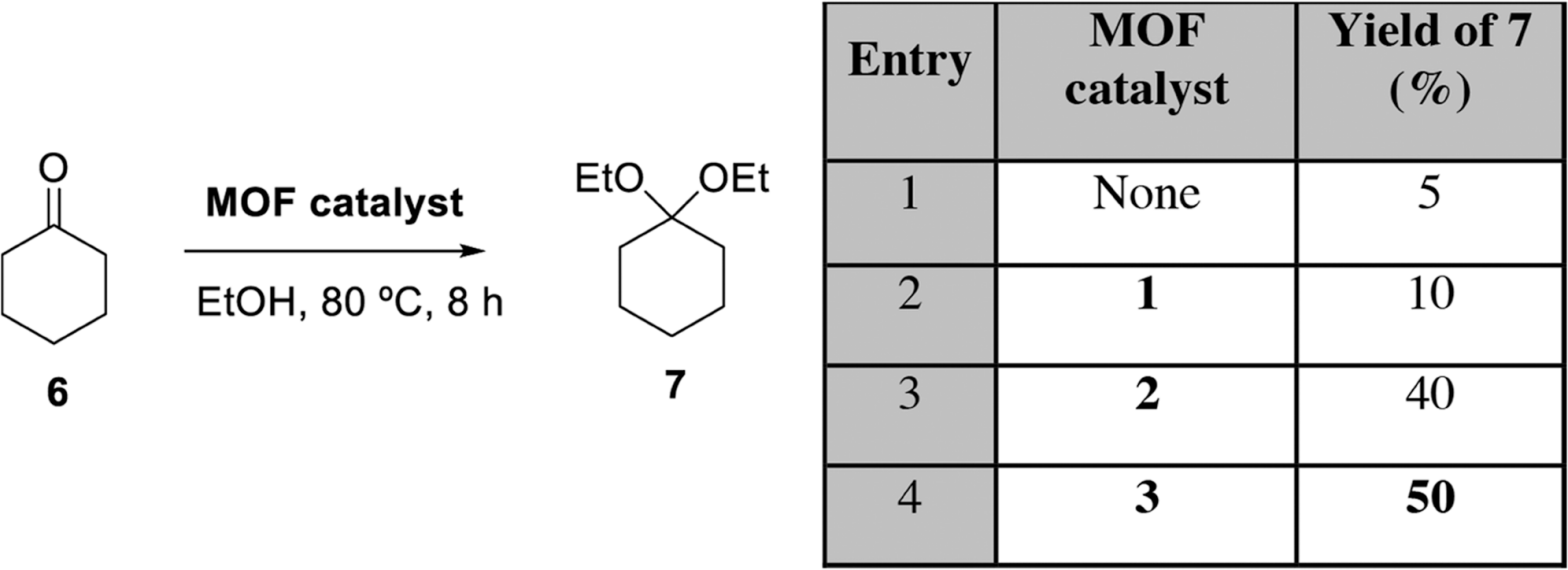
Results for the MOF-catalyzed hemiketalization reaction of cyclohexanone **6** with ethanol. Selectivity to **7** is 100% (conversion equals yield). GC yields.

Ketones are more unreactive than aldehydes in ketalization reactions; however, they were also tested with the MOF catalysts. Figure 5 shows the catalytic results for cyclohexanone **6**. In accordance with the lower reactivity of ketones, a higher reaction temperature (80 °C) was needed to get significant conversions, and EtOH instead of MeOH was used as a nucleophile to avoid overpressure in the reaction. The results show that again MTV-MOF **3** was the best catalyst, although with just a slight difference from MOF **2** (compare entries 3 and 4), to afford 50% of product **7** with complete selectivity. Thus, MTV-MOF **3** can be considered as a general catalyst for the hemiketalization reaction of simple carbonyl compounds (aldehydes and ketones).

## Conclusions

Here, we have presented the synthesis and crystal structure of a novel component of the oxamidato-based ligands derived from the amino acid 3D MTV-MOFs family, and we have reported the influence of the functionalization of the channels, with one or two types of distinct functional groups decorating the channels, on the hemiketalization reaction of different simple carbonyl compounds. On the basis of the obtained results, we can confirm that the heterogeneity present in MTV-MOF **3** is translated well into a more efficient catalytic behavior on the reactions under study. Thus, these results also illustrate the potential of MTV-MOFs to somehow mimic natural nonacidic enzymes and to uncover novel catalytic mechanisms not accessible with traditional microporous materials or single-component MOFs. Nevertheless, further work will be needed to fully uncover the key role that the distinct functional groups play in MTV-MOFs, and how the subtle modification of their proportions and/or spatial arrangement has a massive influence on their functionality, in a similar manner as it occurs in natural enzymes.

## Experimental Section

### Materials

Reagents were obtained from commercial sources (Merck-Aldrich) and used without further purification unless otherwise indicated. Anhydrous solvents were obtained from a resin-exchanger apparatus. Reactions were performed in conventional round-bottomed flasks or sealed vials equipped with a magnetic stirrer. All of the products were characterized by gas chromatography-mass spectrometry (GC-MS). {Sr^II^Cu_6_^II^[(*S*,*S*)-serimox]_3_(OH)_2_(H_2_O)}·38H_2_O (**1**) and {Sr^II^Cu_6_^II^[(*S*,*S*)-mecysmox]_3_(OH)_2_(H_2_O)}·15H_2_O (**2**) were prepared following a previously reported procedure.

### Physical Techniques

Elemental (C, H, S, N) analyses were performed at the Microanalytical Service of the Universitat de València. FT-IR spectra were recorded on a Perkin Elmer 882 spectrophotometer as KBr pellets. The thermogravimetric analysis was performed on crystalline samples under a dry N_2_ atmosphere with a Mettler Toledo TGA/STDA 851^e^ thermobalance operating at a heating rate of 10 °C min^–1^.

### Preparation of {Sr^II^Cu_6_^II^[(*S*,*S*)-serimox]_1.50_[(*S*,*S*)-mecysmox]_1.50_(OH)_2_(H_2_O)}·12H_2_O (**3**)

Suitable well-shaped prisms of **3** for SCXRD were synthesized by slow diffusion in H-shaped tubes of aqueous solutions containing stoichiometric amounts of an equimolar mixture of (Me_4_N)_2_{Cu_2_[(*S*,*S*)-serimox](OH)_2_}·5H_2_O (0.118 g, 0.18 mmol) and (Me_4_N)_2_{Cu_2_[(*S*,*S*)-mecysmox](OH)_2_}·5H_2_O (0.129 g, 0.18 mmol) in one arm and Sr(NO_3_)_2_ (0.025 g, 0.12 mmol) in the other. They were isolated by filtration on paper and air-dried. Alternatively, a gram-scale procedure can also be successfully followed by mixing greater amounts of (Me_4_N)_2_{Cu_2_[(*S*,*S*)-serimox](OH)_2_}·5H_2_O (3.96 g, 6.0 mmol) and (Me_4_N)_2_{Cu_2_[(*S*,*S*)-mecysmox](OH)_2_}·5H_2_O (4.32 g, 6 mmol) in water (50 mL) and adding dropwise another aqueous solution of Sr(NO_3_)_2_ (0.846 g, 4.0 mmol). After allowing the final mixture of reaction to react, under stirring for 6 h, a green polycrystalline powder was isolated by filtration and characterized by C, H, S, N analysis to obtain the final formula of {Sr^II^Cu_6_^II^[(*S*,*S*)-serimox]_1.50_[(*S*,*S*)-mecysmox]_1.50_(OH)_2_(H_2_O)}·12H_2_O. Anal. calcd for **3**: C_27_Cu_6_SrS_3_H_66_N_6_O_40_ (1679.92): C, 19.30; H, 3.96; S, 5.73; N, 5.00%. Found: C, 19.63; H, 3.91; S, 5.78; N, 5.03%. IR (KBr): 1605 and 1602 cm^–1^ (C=O).

### Gas Adsorption

The N_2_ adsorption–desorption isotherms at 77 K were obtained for the polycrystalline samples of **3** with a BELSORP-mini-X instrument. The samples were first activated with methanol and then evacuated at 348 K for 16 h under 10^–6^ Torr prior to their analysis.

### X-ray Powder Diffraction Measurements

A polycrystalline sample of **3** was introduced into a 0.5 mm borosilicate capillary prior to being mounted and aligned on an Empyrean PANalytical powder diffractometer using Cu Kα radiation (λ = 1.54056 Å). Five repeated measurements were collected at room temperature (2θ = 2–45°) and merged in a single diffractogram. A polycrystalline sample of **3** was also measured after catalysis following the same procedure.

### X-ray Crystallographic Data Collection and Structure Refinement

Crystal of **3** with 0.16 mm × 0.14 mm × 0.12 mm as dimensions was selected and mounted on a MiTeGen MicroMount in Paratone oil and very quickly placed on a liquid nitrogen stream cooled at 90 K, to avoid the possible degradation upon dehydration or exposure to air. Diffraction data were collected on a Bruker-Nonius X8APEXII CCD area detector diffractometer using graphite-monochromated Mo Kα radiation (λ = 0.71073 Å). The data were processed through SAINT reduction and SADABS multiscan absorption software. The structure was solved with the SHELXS structure solution program using the Patterson method. The model was refined with version 2018/3 of SHELXL against *F*^2^ on all data by full-matrix least squares.

In the refinement of **3**, all non-hydrogen atoms were refined anisotropically except for some highly dynamically disordered atoms of methylcysteine and serine arms and solvent water molecules. The use of some bond length restraints, applied on atoms belonging to highly dynamic moieties, has been reasonably imposed and related to the expected thermal motion, likely depending on the large size of the huge cages of the frameworks (DFIX and ISOR). For instance, EADP for a group of atoms of the fragments expected to have essentially similar ADPs has been applied. All of the hydrogen atoms of the ligand were set in a calculated position and refined isotropically using the riding model. Hydrogen atoms on thermally disordered solvent water molecules were neither found nor calculated.

As stated in the main text, the oxamidato-bridged dicopper(II) units of {Cu_2_^II^[(*S*,*S*)-serimox]} and {Cu_2_^II^[(*S*,*S*)-mecysmox]}, added in a 1:1 ratio in **3**, exhibit a statistical disorder in the crystal structure, where a very similar percentage of serimox and mecysmox leads to a completely superimposed snapshot of mixed {Cu_2_^II^[(S,S)-mecysmox/serimox]} dimers (see the inset of Figure 1a).

A summary of the crystallographic data and structure refinement for the crystal structure of **3** is given in Table S1. The comments for alerts A and B are described in the CIF using the validation reply form (vrf). CCDC reference number is 2241172.

The final geometrical calculations on free voids and the graphical manipulations were carried out with PLATON^42^ implemented in WinGX^43^ and CRYSTAL MAKER programs,^44^ respectively.

### Typical Catalytic Reaction Procedure

MOFs **1** and **2** or MTV-MOF **3** (25 mg, 100 wt %) were placed in a 2 mL vial equipped with a magnetic stir bar, and the corresponding amount of MeOH (1 mL) was added. Then, the corresponding aldehyde (for example, 26 μL, 0.24 mmol, for **4**) was added via a syringe at room temperature. The mixture was sealed and magnetically stirred in a preheated oil bath at 60 °C for 8 h. For kinetic experiments, individual reactions were placed for each point and aliquots of 0.125 mL were periodically taken. After that, the reaction mixture was poured into AcOEt (1 mL), *n*-dodecane (11 mL, 0.05 mmol) was added as an external standard, and the mixture was passed through a filter syringe and subjected to GC and GC-MS analysis.

## Supporting Information Available

Preparation and physical characterization data, crystallographic refinement details for **3**, and catalytic results (Scheme S1, Figures S1–S6, and Table S1) (PDF)
