# Supplementary material for: Exploring the Role of Amino Acid-Derived Multivariate Metal–Organic Frameworks as Catalysts in Hemiketalization Reactions
Source: Inorg Chem. 2023 Apr 28;62(19):7353–9. doi: 10.1021/acs.inorgchem.3c00495 (PMC10189732; doi:10.1021/acs.inorgchem.3c00495)
Supplement: Supplementary file 1 — ic3c00495_si_001.pdf [file ic3c00495_si_001.pdf]

**Supporting Information** (SI) for the manuscript:

# Exploring the Role of Amino Acid-Derived Multivariate-Metal-Organic Frameworks as Catalyst in (Hemi)-Ketalization Reactions

Cristina Negro,[a‡] Sergio Sanz-Navarro,[b‡] Antonio Leyva-Pérez,*[b] Donatella Armentano,*[c] Jesús Ferrando-Soria,*[a] and Emilio Pardo*[a]

[a]Instituto de Ciencia Molecular (ICMol), Universidad de Valencia, 46980 Valencia, Spain.

[b] Instituto de Tecnología Química (UPV–CSIC), Universidad Politècnica de València–Consejo Superior de Investigaciones Científicas, Avda. de los Naranjos s/n, 46022 Valencia, Spain.

[c]Dipartimento di Chimica e Tecnologie Chimiche (CTC), Università della Calabria, Rende 87036, Italy.

Corresponding authors emails: Antonio Leyva-Pérez (anleyva@itq.upv.es), Donatella Armentano (donatella.armentano@unical.it), Jesús Ferrando-Soria (jesus.ferrando@uv.es) and Emilio Pardo (emilio.pardo@uv.es).

**Materials.** Reagents were obtained from commercial sources (Merck-Aldrich) and used without further purification otherwise indicated. Anhydrous solvents were obtained from a resin-exchanger apparatus. Reactions were performed in conventional round-bottomed flasks or sealed vials equipped with a magnetic stirrer. All the products were characterized by gas chromatography-mass spectrometry (GC-MS). $\{Sr^{II}Cu^{II}_6[(S,S)$-serimox$]_3(OH)_2(H_2O)\}$ · 38 $H_2O$ (**1**) and $\{Sr^{II}Cu^{II}_6[(S,S)$-Mecysmox$]_3(OH)_2(H_2O)\}$ · 15 $H_2O$ (**2**) were prepared following a previously reported procedure.[1,2]

**Physical Techniques.** Elemental (C, H, S, N) analyses were performed at the Microanalytical Service of the Universitat de València. FT–IR spectra were recorded on a Perkin-Elmer 882 spectrophotometer as KBr pellets. The thermogravimetric analysis was performed on crystalline samples under a dry $N_2$ atmosphere with a Mettler Toledo TGA/STDA 851$^e$ thermobalance operating at a heating rate of 10 ºC min$^{-1}$.

**Preparation of $\{Sr^{II}Cu^{II}_6[(S,S)$-serimox$]_{1.50}[(S,S)$-Mecysmox$]_{1.50}(OH)_2(H_2O)\}$ · 12 $H_2O$ (3).** Suitable well-shaped prisms of **3** for SCXRD were synthetized by slow diffusion in H-shaped tubes of aqueous solutions containing stoichiometric amounts of an equimolar mixture of $(Me_4N)_2\{Cu_2[(S,S)$-serimox$](OH)_2\}$ · $5H_2O$ (0.118 g, 0.18 mmol) and $(Me_4N)_2\{Cu_2[(S,S)$-Mecysmox$](OH)_2\}$ · $5H_2O$ (0.129 g, 0.18 mmol) in one arm and $Sr(NO_3)_2$ (0.025 g, 0.12 mmol) in the other. They were isolated by filtration on paper and air-dried. Alternatively, a gram-scale procedure can be also successfully followed by mixing greater amounts of $(Me_4N)_2\{Cu_2[(S,S)$-serimox$](OH)_2\}$ · $5H_2O$ (3.96 g, 6.0 mmol) and $(Me_4N)_2\{Cu_2[(S,S)$-Mecysmox$](OH)_2\}$ · $5H_2O$ (4.32 g, 6 mmol) in water (50 mL), and dropwise adding another aqueous solution of $Sr(NO_3)_2$ (0.846 g, 4.0 mmol). After allowing to react the final mixture of reaction, under stirring, for 6 h, a green polycrystalline powder was isolated by filtration and

characteirsed by C, H, S, N analysis to give the final formula of $\{Sr^{II}Cu^{II}_6[(S,S)$-serimox$]_{1.50}[(S,S)$-Mecysmox$]_{1.50}(OH)_2(H_2O)\}$ · 12 $H_2O$. Anal. Calcd for **3**: $C_{27}Cu_6SrS_3H_{66}N_6O_{40}$ (1679.92): C, 19.30; H, 3.96; S, 5.73; N, 5.00 %. Found: C, 19.63; H, 3.91; S, 5.78; N, 5.03%. IR (KBr):  = 1605 and 1602 $cm^{-1}$ (C=O)

**Gas adsorption.** The $N_2$ adsorption-desorption isotherms at 77 K were carried out on polycrystalline samples of **3** with a BELSORP-mini-X instrument. Samples were first activated with methanol and then evacuated at 348 K during 16 hours under $10^{-6}$ Torr prior to their analysis.

**X-ray Powder Diffraction Measurements.** Polycrystalline samples of **3** was introduced into a 0.5 mm borosilicate capillar prior to being mounted and aligned on a Empyrean PANalytical powder diffractometer, using Cu Kα radiation (λ = 1.54056 Å). Five repeated measurements were collected at room temperature (2θ = 2–45°) and merged in a single diffractogram. A polycrystalline sample of **3** was also measured after catalysis following the same procedure.

**X-ray Crystallographic Data Collection and Structure Refinement.** Crystal of **3** with 0.16 x 0.14 x 0.12 mm as dimensions was selected and mounted on a MiTeGen MicroMount in Paratone oil and very quickly placed on a liquid nitrogen stream cooled at 90 K, to avoid the possible degradation upon dehydration or exposure to air. Diffraction data were collected on a Bruker-Nonius X8APEXII CCD area detector diffractometer using graphite-monochromated Mo-$K_\alpha$ radiation (λ = 0.71073 Å), The data were processed through SAINT reduction and SADABS multi-scan absorption software.[3] The structure was solved with the SHELXS structure solution program, using the Patterson method. The model was refined with version 2018/3 of SHELXL against $F^2$ on all data by full-matrix least squares.[4,5]

In the refinement of **3**, all non-hydrogen atoms were refined anisotropically except some highly dynamically disordered atoms of methylcysteine and serine arms and solvent water molecules. The use of some bond lengths restraints, applied on atoms belonging to highly dynamic moieties, has been reasonably imposed and related to the expected thermal motion, likely depending on the large size of the huge cages of the frameworks (DFIX and ISOR). For instance, EADP for group of atoms of the fragments expected to have essentially similar ADPs have been applied. All the hydrogen atoms of the ligand were set in calculated position and refined isotropically using the riding model. Hydrogen atoms on thermally disordered solvent water molecules were neither found nor calculated.

As stated in main text, the oxamidato-bridged dicopper(II) units of {$Cu^{II}_2$[(*S*,*S*)-serimox]} and {$Cu^{II}_2$[(*S*,*S*)-mecysmox]}, inserted with a 1:1 ratio in **3**, exhibit a statistically disorder in the crystal structure, where the very similar percentage of serimox and mecysmox leads to a completely superimposed snapshot of mixed {$Cu^{II}_2$[(S,S)-mecysmox/serimox]} dimers (see inset of Figure 1a).

A summary of the crystallographic data and structure refinement for **3** crystal structure is given in Table S1. The comments for the alerts A and B are described in the CIF using the validation reply form (vrf). CCDC reference number is 2241172.

The final geometrical calculations on free voids and the graphical manipulations were carried out with PLATON[6] implemented in WinGX,[7] and CRYSTAL MAKER programs,[8] respectively.

**Catalytic experiments.**

**Leaching test.** MTV-MOF **3** (25 mg, 100 wt%) was placed in a 2 ml vial equipped with a magnetic stir bar, and the corresponding amount of MeOH (1 mL) was added.

Then, benzaldehyde **4** (26 μL, 0.24 mmol) was added via syringe at room temperature. The mixture was sealed and magnetically stirred in a pre–heated oil bath at 60 $^{o}$C. After 30 min reaction time, the solid catalyst was filtered off and the filtrates were magnetically stirred in a pre–heated oil bath at 60 $^{o}$C for 7 h 30 min, taking periodically aliquots of 0.125 mL to be analysed by GC. The kinetic results were compared with the reaction containing MTV-MOF **3**.

**Reuses of the solid catalyst**. The general reaction procedure above was followed. After the reaction time, the solid catalyst was recovered by filtration and washed with hexane. After drying, MTV-MOF **3** was weighted and benzaldehyde **4** added in proportional amount to keep the initial relative molar ratios.

**Table S1.** Summary of Crystallographic Data for **3**.

| Compound | **3** |
|---|---|
| Formula | $C_{27}Cu_6SrS_3H_{66}N_6O_{40}$ |
| $M$ (g mol$^{-1}$) | 1679.89 |
| λ (Å) | 0.71073 |
| Crystal system | Hexagonal |
| Space group | $P6_3$ |
| $a$ (Å) | 17.9936(10) |
| $c$ (Å) | 13.0313(8) |
| $V$ (Å$^3$) | 3653.9(5) |
| $Z$ | 2 |
| $\rho_{calc}$ (g cm$^{-3}$) | 1.527 |
| $\mu$ (mm$^{-1}$) | 2.608 |
| $T$ (K) | 293 |
| $\theta$ range for data collection (°) | 2.614to 26.279 |
| Completeness to $\theta$ = 25.0 | 100% |
| Measured reflections | 66317 |
| Unique reflections (Rint) | 4938 (0.0632) |
| Observed reflections [$I > 2\sigma(I)$] | 3830 |
| Goof | 1.054 |
| Absolute structure parameter (Flack) | 0.046(5) |
| $R^a$ [$I > 2\sigma(I)$] (all data) | 0.0670 (0.0870) |
| $wR^b$ [$I > 2\sigma(I)$] (all data) | 0.1982 (0.2179) |
| Largest diff. peak and hole | 1.496 and -0.566 e.Å$^{-3}$ |

$^a$ $R = \sum(|F_o| - |F_c|)/\sum|F_o|$. $^b$ $wR = [\sum w(|F_o| - |F_c|)^2/\sum w|F_o|^2]^{1/2}$. $^c$The poor quality of the crystals of **2** allowed only cell parameters determination.

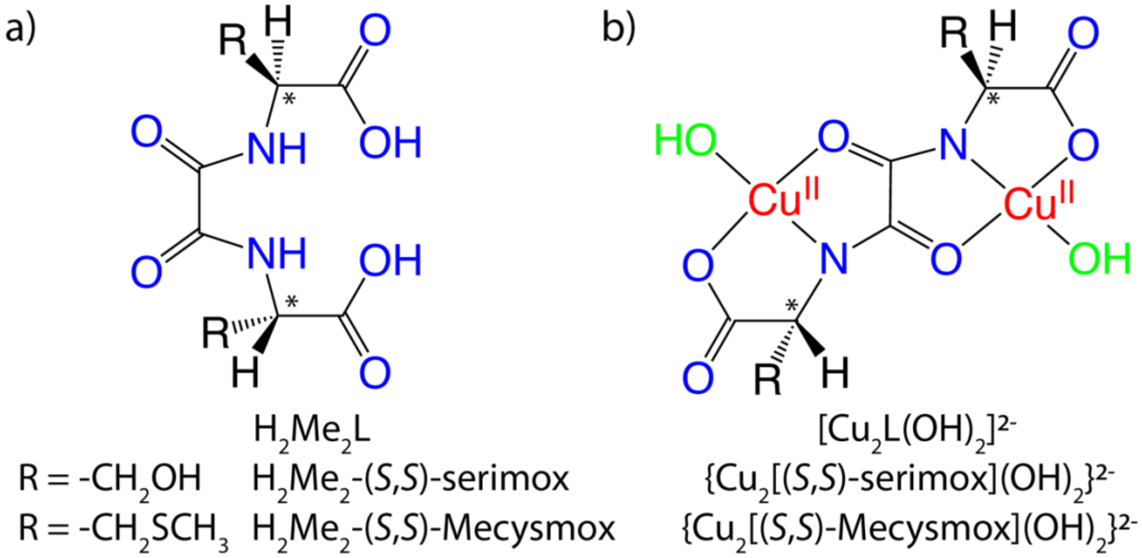


**Scheme S1.** Chemical structures of the chiral bis(amino acid)oxalamide ligands (a), highlighting the potential coordination sites and chiral centers (*) and the corresponding dianionic bis(hydroxo)dicopper(II) complexes (b).

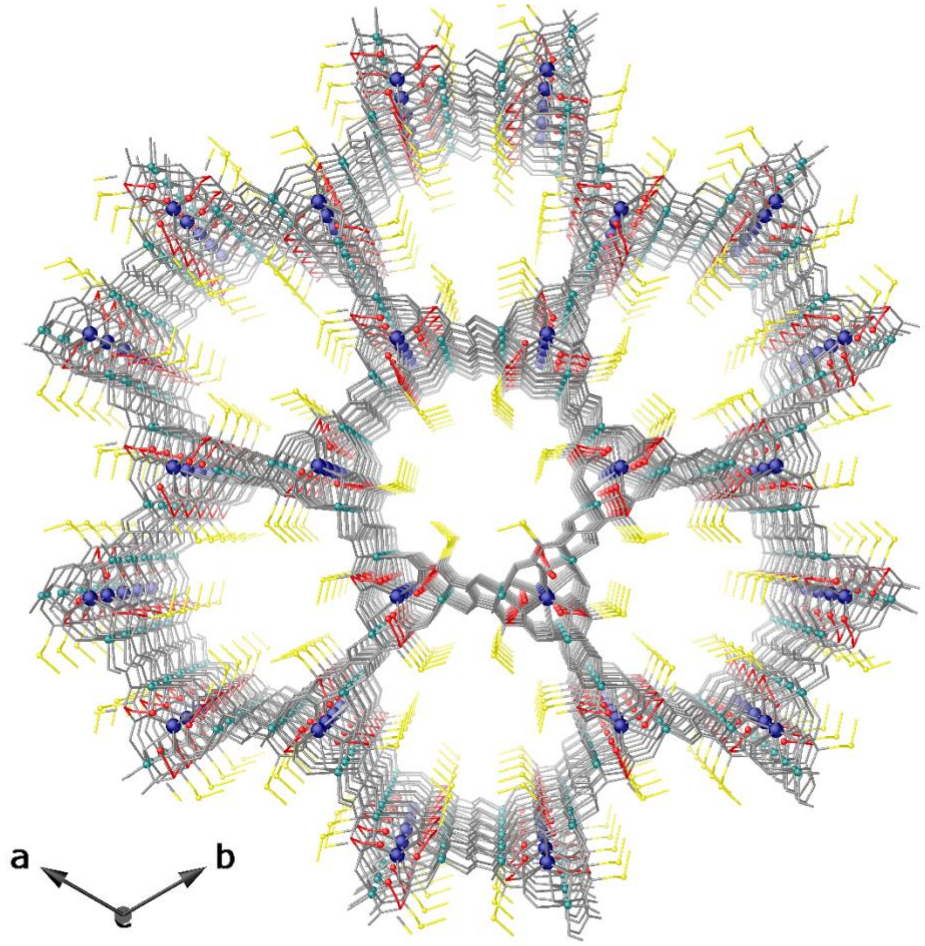


**Figure S1.** Perspective view of the crystal of **3** along *c* crystallographic axes Copper(II) and strontium(II) ions from the network are represented as cyan and blue spheres, respectively. Oxygen and sulfur atoms from the residues are shown as red and yellow spheres, respectively. The organic ligands are represented as gray sticks, with the exception of *L*-serine (−$CH_2OH$) and *L*-methylcysteine (−$CH_2SCH_3$) residues, which are represented as red and yellow sticks, respectively.

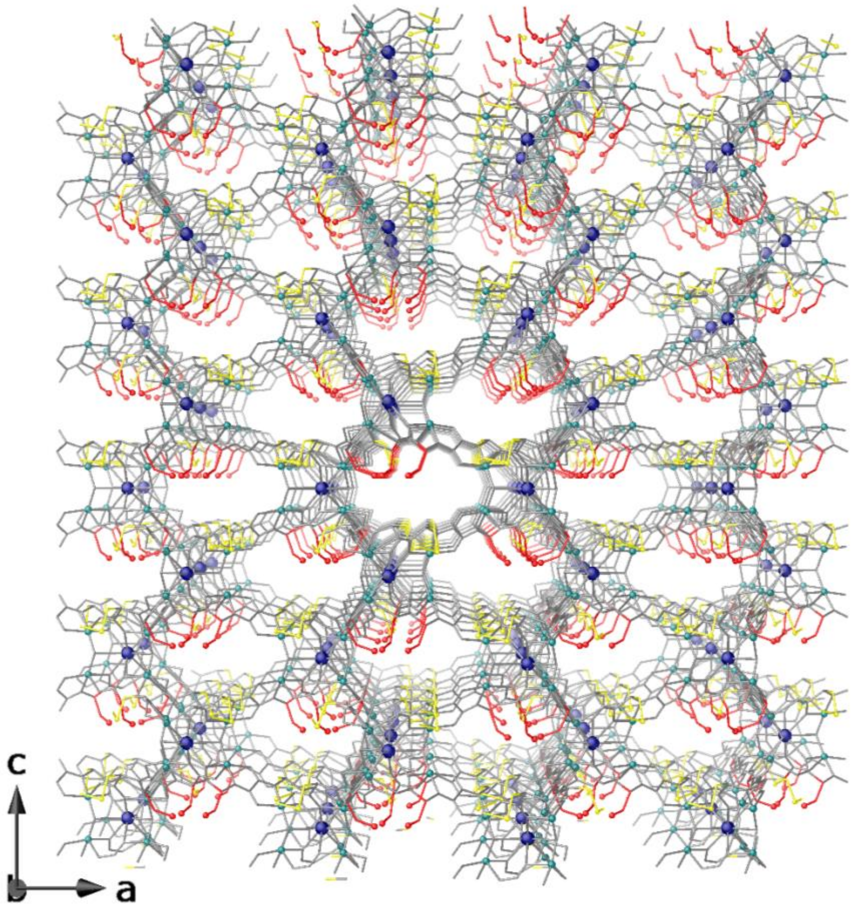


**Figure S2.** Perspective view of the crystal of **3** along *b* crystallographic axes. Copper(II) and strontium(II) ions from the network are represented as cyan and blue spheres, respectively. Oxygen and sulfur atoms from the residues are shown as red and yellow spheres, respectively. The organic ligands are represented as gray sticks, with the exception of *L*-serine ($-CH_2OH$) and *L*-methylcysteine ($-CH_2SCH_3$) residues, which are represented as red and yellow sticks, respectively.

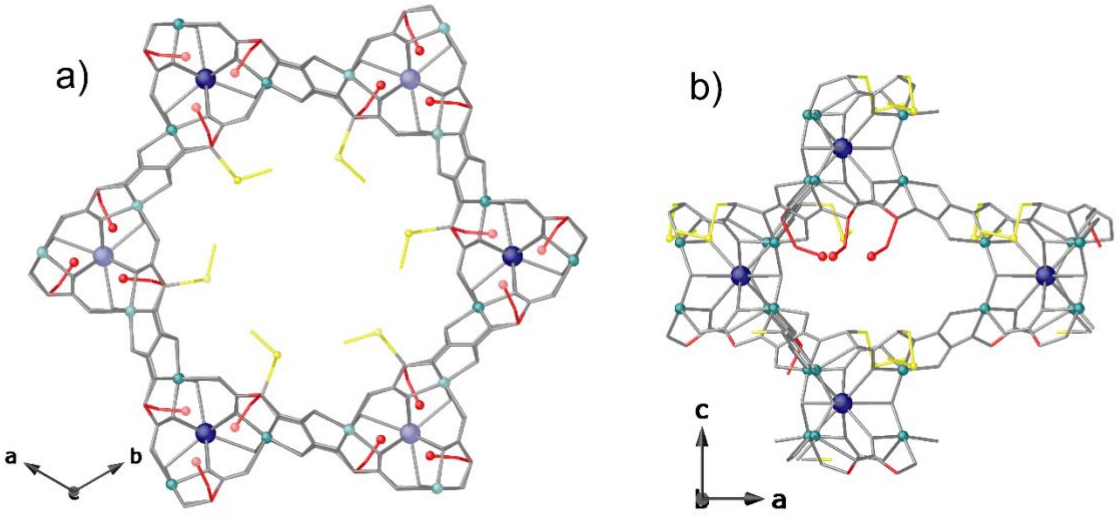


**Figure S3.** Details of conformations and distribution of *L*-serine ($-CH_2OH$) and *L*-methylcysteine ($-CH_2SCH_3$) residues a) along *c* and b) *b* crystallographic axis, respectively, pointing towards big pores and smallest voids in **3**. The organic ligands are represented as gray sticks, with the exception of *L*-serine ($-CH_2OH$) and *L*-methylcysteine ($-CH_2SCH_3$) residues, which are represented as red and yellow sticks, respectively.

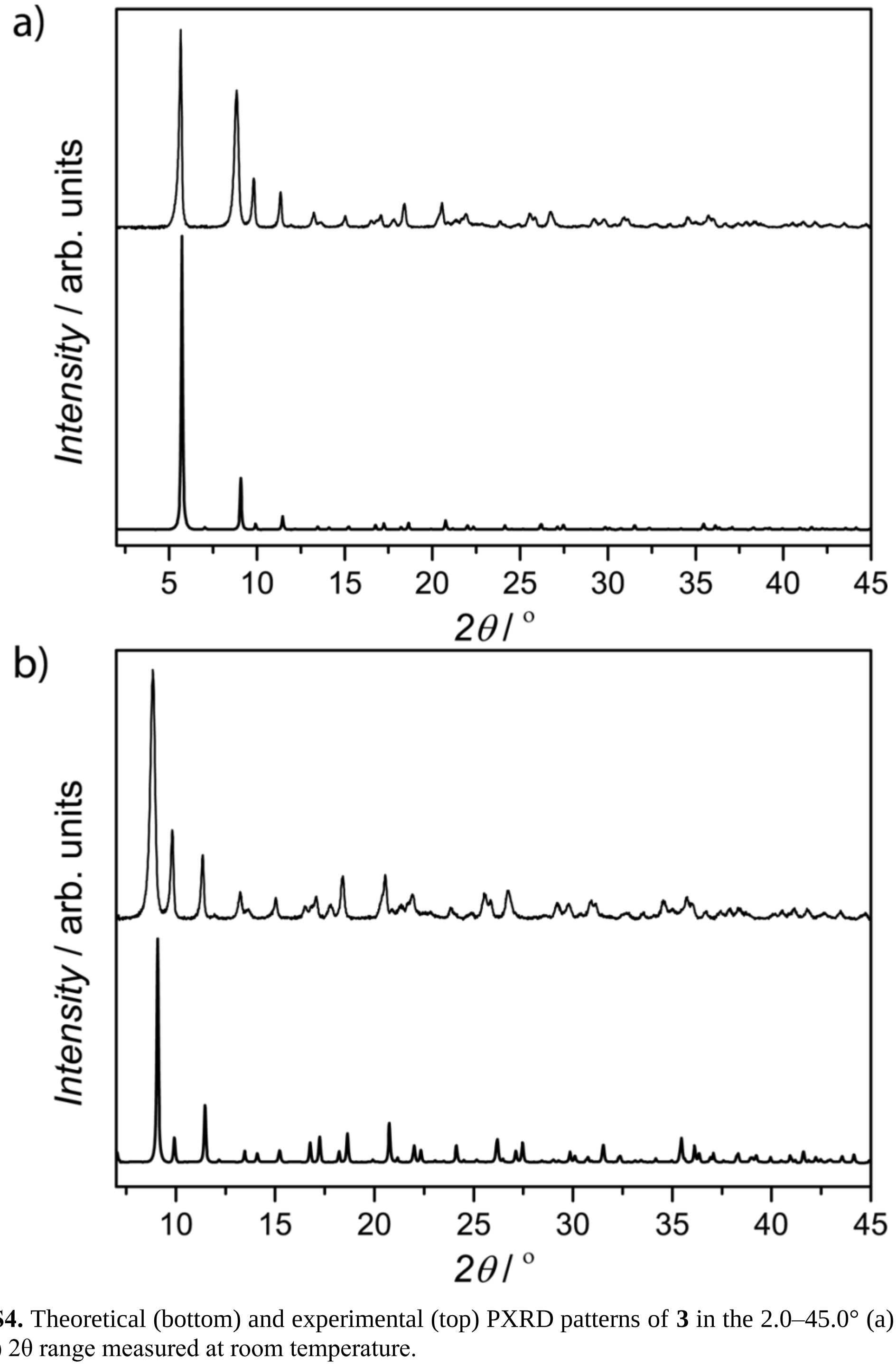


**Figure S4.** Theoretical (bottom) and experimental (top) PXRD patterns of **3** in the 2.0–45.0° (a) and 7.0–45.0° (b) 2θ range measured at room temperature.

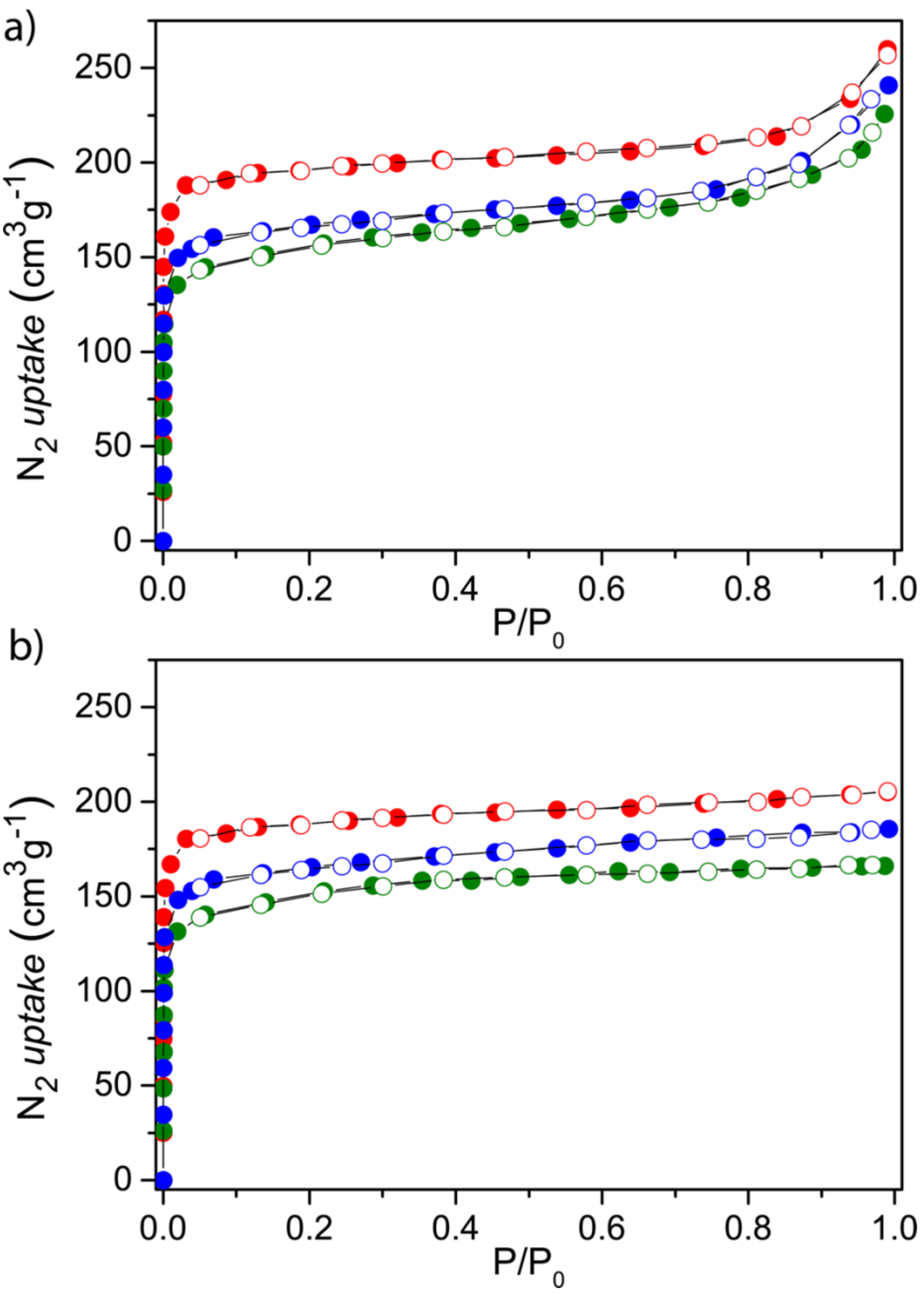


**Figure S5.** (a) $N_2$ (77 K) adsorption isotherms for the activated compounds **1** (red), **2** (green) and **3** (blue). (b) $N_2$ (77 K) adsorption isotherms for the activated compounds **1** (red), **2** (green) and **3** (blue) after catalytic experiments. Filled and empty symbols indicate the adsorption and desorption isotherms, respectively. The samples were activated at 70 °C under reduced pressure for 16 h prior to carry out the sorption measurements.

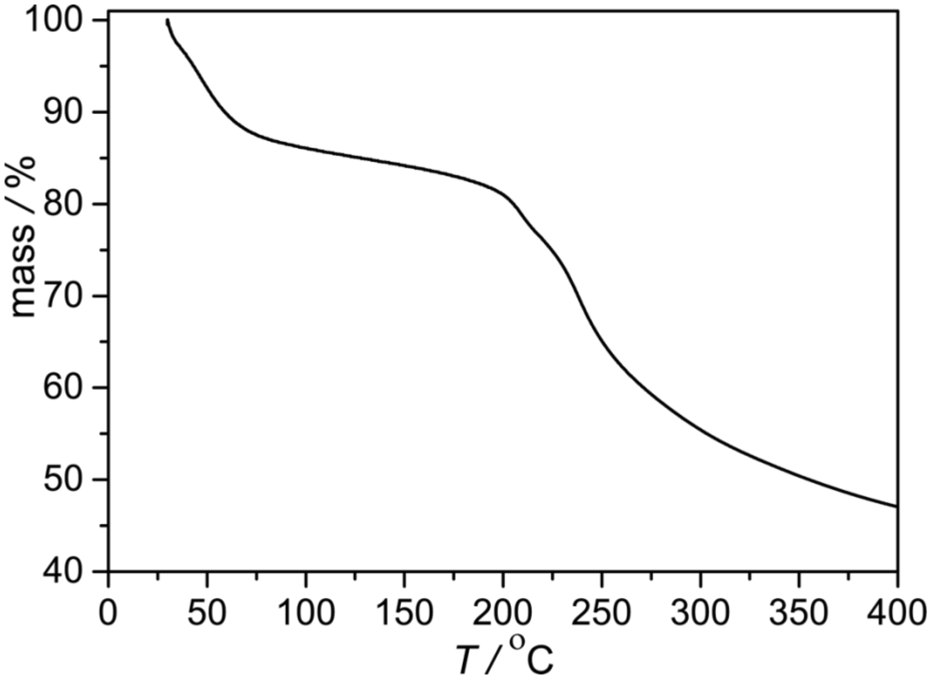


**Figure S6.** Thermo-Gravimetric analysis (TGA) of **3** under dry $N_2$ atmosphere.

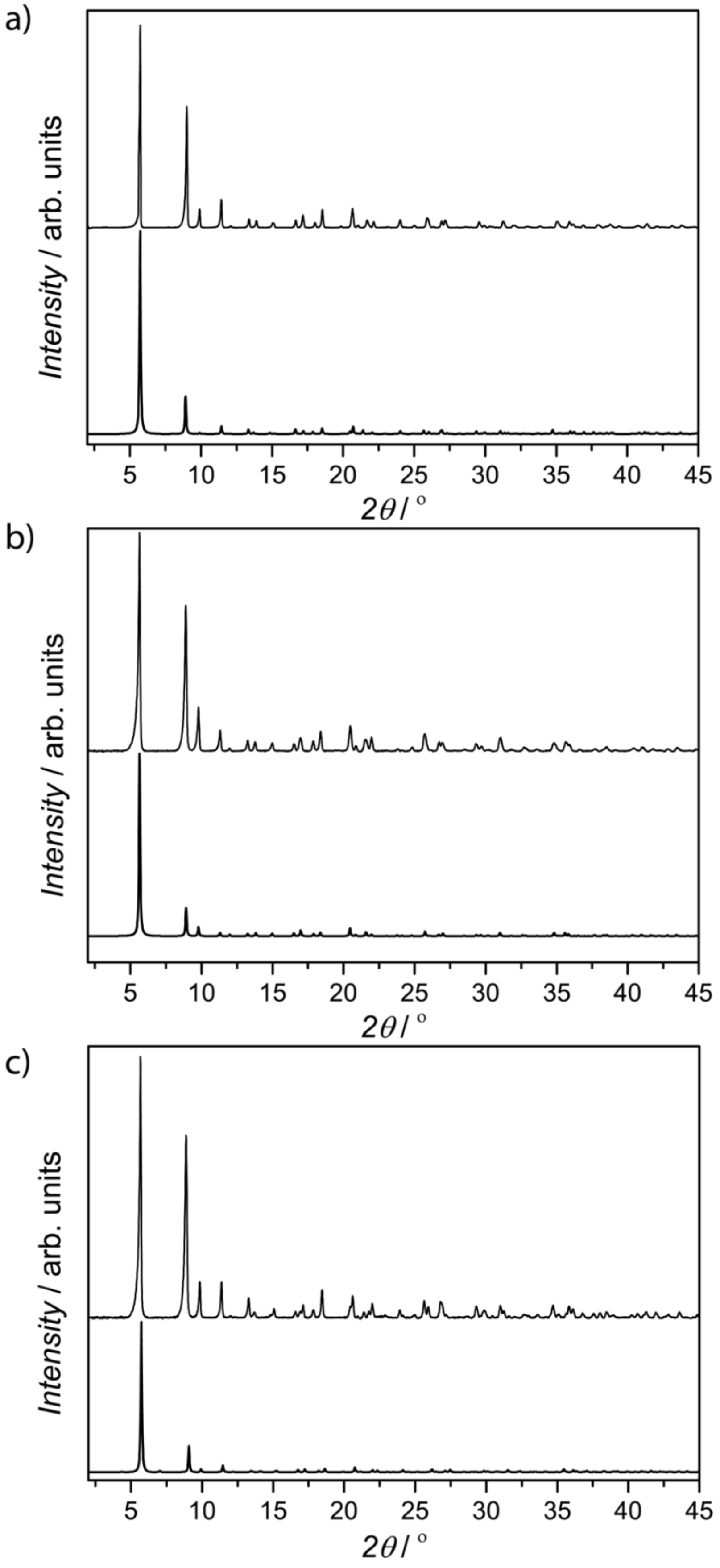


**Figure S7.** Theoretical (bottom) and experimental (top) PXRD patterns of **1** (a), **2** (b) and **3** (c) after catalysis in the 2.0–45.0° 2θ range measured at room temperature.

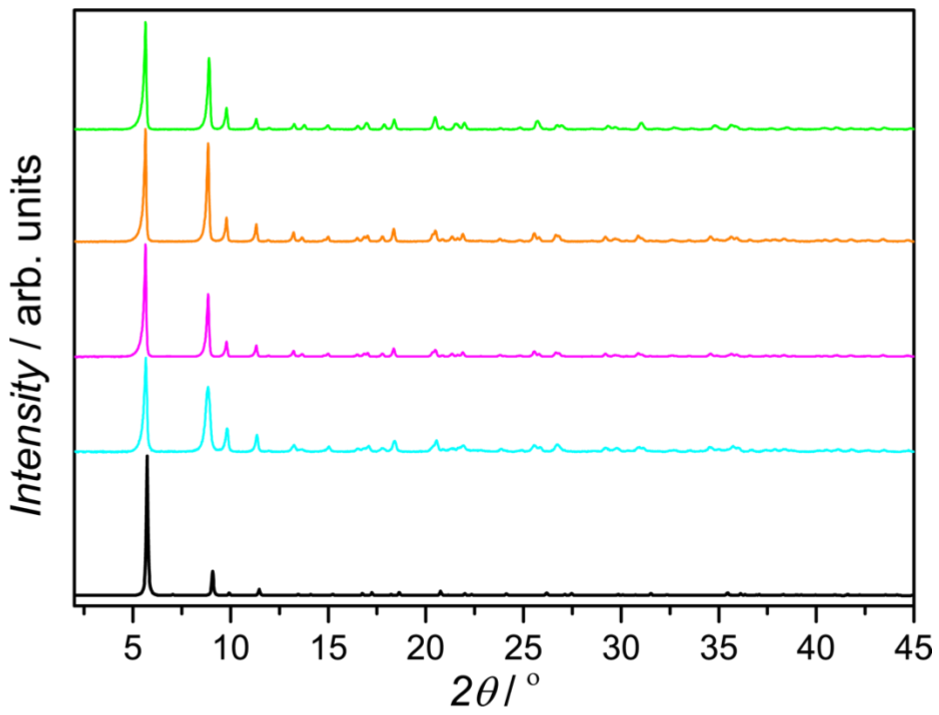


**Figure S8.** Theoretical (bottom) and experimental (top) PXRD patterns of **3** after being immersed in hot water (cyan), dimethylformamide (purple), methanol (orange) and acetonitrile (light green) in the 2.0–45.0° 2θ range measured at room temperature.

## References


1. (a) Mon, M.; Bruno, R.; Ferrando-Soria, J.; Bartella, L.; Di Donna, L.; Talia, M.; Lappano, R.; Maggiolini, M.; Armentano, D.; Pardo, E. Crystallographic Snapshots of Host–Guest Interactions in Drugs@metal–Organic Frameworks: Towards Mimicking Molecular Recognition Processes. *Mater. Horizons* **2018**, *5*, 683–690. (b) Mon, M.; Bruno, R.; Elliani, R.; Tagarelli, A.; Qu, X.; Chen, S.; Ferrando-Soria, J.; Armentano, D.; Pardo, E. Lanthanide Discrimination with Hydroxyl-Decorated Flexible Metal–Organic Frameworks. *Inorg. Chem.* **2018**, *57*, 13895–13900

2. Tiburcio, E.; Greco, R.; Mon, M.; Ballesteros-Soberanas, J.; Ferrando-Soria, J.; López-Haro, M.; Hernández-Garrido, J. C.; Oliver-Meseguer, J.; Marini, C.; Boronat, M.; Armentano, D.; Leyva-Pérez, A.; Pardo, E. Soluble/MOF-Supported Palladium Single Atoms Catalyze the Ligand-, Additive-, and Solvent-Free Aerobic Oxidation of Benzyl Alcohols to Benzoic Acids. *J. Am. Chem. Soc.* **2021**, *143*, 2581–2592.

3. Sheldrick, G. M. *SADABS Program for Absorption Correction*, ver. 2.10; Analytical X-ray Systems: Madison, WI, 1998.

4. Sheldrick, G. M. A short history of SHELX. *Acta. Crystallogr., Sect. A: Found. Crystallogr*. **2008**, *64*, 112–122.

5. Sheldrick, G. M. Crystal structure refinemnet with SHELXL. *Acta. Crystallogr., Sect. C: Struct. Chem.* **2015**, *71*, 3–8.

6. Parsons, S.; Flack, H. D.; Wagner, T. Use of intensity quotients and differences in absolute structure refinement. *Acta Crystallogr., Sect. B*: *Struct. Sci.*, *Cryst. Eng. Mater.* **2013**, *69*, 249–259.

7. (a) Farrugia, L. J. WinGX suite for small-molecule single-crystal crystallography. *J. Appl. Crystallogr.* **1999**, *32*, 837–838. (b) Farrugia, L. J. WinGX and ORTEP for Windows: An Update. *J. Appl. Crystallogr.* **2012**, *45*, 849–854.

8. Palmer, D. C. Zeitschrift fur Krist. *Z. Kristallogr. – Cryst. Mater.* **2015**, *230*, 559–572.
